# 5-(3-Pyrid­yl)-1,3,4-thia­diazol-2-amine

**DOI:** 10.1107/S1600536809019564

**Published:** 2009-05-29

**Authors:** Yao Wang, Rong Wan, Feng Han, Peng Wang

**Affiliations:** a Department of Applied Chemistry, College of Science, Nanjing University of Technology, No. 5 Xinmofan Road, Nanjing 210009, People’s Republic of China

## Abstract

The title compound, C_7_H_6_N_4_S, was synthesized by reacting pyridine-3-carboxylic acid and thio­semicarbazide. The dihedral angle between the planes of the thia­diazole and pyridine rings is 32.42 (14)°. In the crystal structure, inter­molecular N—H⋯N inter­actions link the mol­ecules into a three-dimensional network, forming *R*_2_^2^(8) ring motifs. π–π contacts between thia­diazole rings [centroid–centroid distance = 3.666 (1) Å] may further stabilize the structure.

## Related literature

For general background, see: Nakagawa *et al.* (1996 ▶); Wang *et al.* (1999 ▶). For a related structure, see: Wang *et al.* (2009 ▶). For bond-length data, see: Allen *et al.* (1987 ▶). For ring-motifs, see: Bernstein *et al.* (1995 ▶).
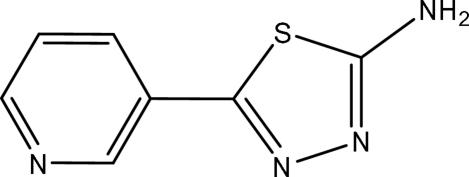


## Experimental

### 

#### Crystal data

C_7_H_6_N_4_S
*M* *_r_* = 178.22
Monoclinic, 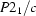

*a* = 11.066 (2) Å
*b* = 7.2380 (14) Å
*c* = 11.271 (2) Å
β = 116.79 (3)°
*V* = 805.9 (3) Å^3^
*Z* = 4
Mo *K*α radiation
μ = 0.35 mm^−1^
*T* = 294 K
0.10 × 0.05 × 0.05 mm

#### Data collection

Enraf–Nonius CAD-4 diffractometer
Absorption correction: ψ scan (North *et al.*, 1968 ▶) *T*_min_ = 0.966, *T*_max_ = 0.983
1542 measured reflections
1464 independent reflections
1220 reflections with *I* > 2σ(*I*)
*R*_int_ = 0.026
3 standard reflections frequency: 120 min intensity decay: 1%

#### Refinement

*R*[*F* ^2^ > 2σ(*F* ^2^)] = 0.043
*wR*(*F* ^2^) = 0.132
*S* = 1.01
1464 reflections
109 parameters
H-atom parameters constrained
Δρ_max_ = 0.29 e Å^−3^
Δρ_min_ = −0.34 e Å^−3^

### 

Data collection: *CAD-4 Software* (Enraf–Nonius, 1989 ▶); cell refinement: *CAD-4 Software*; data reduction: *XCAD4* (Harms & Wocadlo, 1995 ▶); program(s) used to solve structure: *SHELXS97* (Sheldrick, 2008 ▶); program(s) used to refine structure: *SHELXL97* (Sheldrick, 2008 ▶); molecular graphics: *ORTEP-3 for Windows* (Farrugia, 1997 ▶) and *PLATON* (Spek, 2009 ▶); software used to prepare material for publication: *SHELXL97*.

## Supplementary Material

Crystal structure: contains datablocks global, I. DOI: 10.1107/S1600536809019564/hk2699sup1.cif

Structure factors: contains datablocks I. DOI: 10.1107/S1600536809019564/hk2699Isup2.hkl

Additional supplementary materials:  crystallographic information; 3D view; checkCIF report

## Figures and Tables

**Table 1:** Hydrogen-bond geometry (Å, °)

*D*—H⋯*A*	*D*—H	H⋯*A*	*D*⋯*A*	*D*—H⋯*A*
N4—H4*A*⋯N3^i^	0.86	2.13	2.959 (3)	163
N4—H4*B*⋯N2^ii^	0.86	2.16	3.006 (3)	168

Symmetry codes: (i) 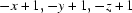; (ii) 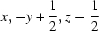.

**Fig. 1.**
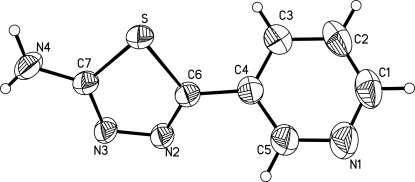
The molecular structure of the title molecule, with the atom-numbering scheme. Displacement ellipsoids at the 50% probability level.

**Fig. 2.**
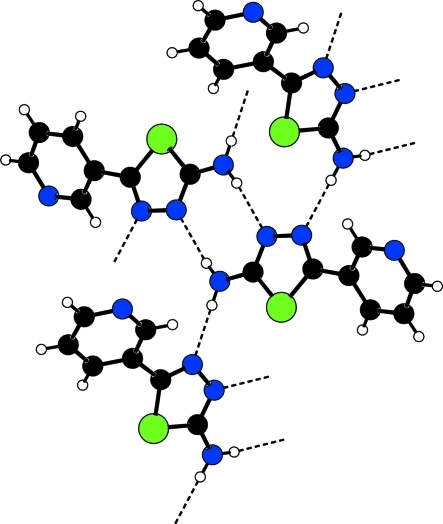
A partial packing diagram of the title compound. Hydrogen bonds are shown as dashed lines.

## supplementary crystallographic information

### Comment

1,3,4-Thiadiazole derivatives represent an interesting class of compounds possessing broad spectrum biological activities (Nakagawa *et al.*, 1996). These compounds are known to exhibit diverse biological effects, such as insecticidal and fungicidal activities (Wang *et al.*, 1999). We report herein the crystal structure of the title compound.

In the molecule of the title compound (Fig 1), the bond lengths (Allen *et al.*, 1987) and angles are within normal ranges, and are in accordance with the corresponding values in 5-(4-pyridyl)-1,3,4-thiadiazol-2-amine (Wang *et al.*, 2009). Rings A (N1/C1–C5) and B (S/N2/N3/C6/C7) are, of course, planar, and they are oriented at a dihedral angle of 32.42 (14)°.

In the crystal structure, intermolecular N—H···N interactions (Table 1) link the molecules into a three-dimensional network forming *R*_2_^2^(8) ring motifs (Bernstein *et al.*, 1995) (Fig. 2), in which they may be effective in the stabilization of the structure. The π–π contact between the thiadiazole rings, Cg2—Cg2^i^, [symmetry code: (i) 1 - x, -y, 1 - z, where Cg2 is centroid of the ring B (S/N2/N3/C6/C7)] may further stabilize the structure, with centroid–centroid distance of 3.666 (1) Å.

### Experimental

For the preparation of the title compound, 3-pyridine carboxylic acid (2 mmol) and thiosemicarbazide (5 mmol) were mixed in a 25 ml flask, and kept in the oil bath at 363 K for 6 h. After cooling, the crude product precipitated and was filted. Crystals of suitable for X-ray analysis were obtained by slow evaporation of an acetone solution.

### Refinement

H atoms were positioned geometrically, with N—H = 0.86 Å (for NH_2_) and C—H = 0.93 Å for aromatic H and constrained to ride on their parent atoms, with U_iso_(H) = 1.2U_eq_(C,N).

### Crystal data

**Table:**

C_7_H_6_N_4_S	*F*(000) = 368
*M**_r_* = 178.22	*D*_x_ = 1.469 Mg m^−^^3^
Monoclinic, *P*2_1_/*c*	Melting point: 550 K
Hall symbol: -P 2ybc	Mo *K*α radiation, λ = 0.71073 Å
*a* = 11.066 (2) Å	Cell parameters from 25 reflections
*b* = 7.2380 (14) Å	θ = 9–12°
*c* = 11.271 (2) Å	µ = 0.35 mm^−^^1^
β = 116.79 (3)°	*T* = 294 K
*V* = 805.9 (3) Å^3^	Block, colourless
*Z* = 4	0.10 × 0.05 × 0.05 mm

### Data collection

**Table:**

Enraf–Nonius CAD-4 diffractometer	1220 reflections with *I* > 2σ(*I*)
Radiation source: fine-focus sealed tube	*R*_int_ = 0.026
graphite	θ_max_ = 25.3°, θ_min_ = 2.1°
ω/2θ scans	*h* = 0→13
Absorption correction: ψ scan (North *et al.*, 1968)	*k* = 0→8
*T*_min_ = 0.966, *T*_max_ = 0.983	*l* = −13→12
1542 measured reflections	3 standard reflections every 120 min
1464 independent reflections	intensity decay: 1%

### Refinement

**Table:**

Refinement on *F*^2^	Primary atom site location: structure-invariant direct methods
Least-squares matrix: full	Secondary atom site location: difference Fourier map
*R*[*F*^2^ > 2σ(*F*^2^)] = 0.043	Hydrogen site location: inferred from neighbouring sites
*wR*(*F*^2^) = 0.132	H-atom parameters constrained
*S* = 1.00	*w* = 1/[σ^2^(*F*_o_^2^) + (0.098*P*)^2^] where *P* = (*F*_o_^2^ + 2*F*_c_^2^)/3
1464 reflections	(Δ/σ)_max_ < 0.001
109 parameters	Δρ_max_ = 0.29 e Å^−^^3^
0 restraints	Δρ_min_ = −0.34 e Å^−^^3^

### Special details

**Table:**

Geometry. All e.s.d.'s (except the e.s.d. in the dihedral angle between two l.s. planes) are estimated using the full covariance matrix. The cell e.s.d.'s are taken into account individually in the estimation of e.s.d.'s in distances, angles and torsion angles; correlations between e.s.d.'s in cell parameters are only used when they are defined by crystal symmetry. An approximate (isotropic) treatment of cell e.s.d.'s is used for estimating e.s.d.'s involving l.s. planes.
Refinement. Refinement of *F*^2^ against ALL reflections. The weighted *R*-factor *wR* and goodness of fit *S* are based on *F*^2^, conventional *R*-factors *R* are based on *F*, with *F* set to zero for negative *F*^2^. The threshold expression of *F*^2^ > σ(*F*^2^) is used only for calculating *R*-factors(gt) *etc*. and is not relevant to the choice of reflections for refinement. *R*-factors based on *F*^2^ are statistically about twice as large as those based on *F*, and *R*- factors based on ALL data will be even larger.

### Fractional atomic coordinates and isotropic or equivalent isotropic displacement parameters (Å^2^)

**Table:**

	*x*	*y*	*z*	*U*_iso_*/*U*_eq_	
S	0.69244 (6)	0.09005 (8)	0.45516 (5)	0.0434 (3)	
N1	0.9145 (3)	−0.2801 (4)	0.8931 (2)	0.0717 (7)	
N2	0.63999 (19)	0.1376 (3)	0.65128 (17)	0.0421 (5)	
N3	0.57605 (19)	0.2824 (3)	0.56552 (18)	0.0441 (5)	
N4	0.5428 (3)	0.3989 (3)	0.3599 (2)	0.0595 (7)	
H4A	0.4944	0.4890	0.3645	0.071*	
H4B	0.5580	0.3880	0.2918	0.071*	
C1	0.9215 (3)	−0.4300 (4)	0.8284 (3)	0.0634 (8)	
H1B	0.9692	−0.5315	0.8782	0.076*	
C2	0.8626 (3)	−0.4435 (4)	0.6931 (3)	0.0628 (8)	
H2B	0.8714	−0.5509	0.6523	0.075*	
C3	0.7896 (3)	−0.2946 (3)	0.6177 (3)	0.0547 (7)	
H3B	0.7483	−0.3004	0.5253	0.066*	
C4	0.7792 (2)	−0.1374 (3)	0.6817 (2)	0.0407 (5)	
C5	0.8440 (3)	−0.1375 (4)	0.8191 (3)	0.0578 (7)	
H5A	0.8380	−0.0315	0.8628	0.069*	
C6	0.7037 (2)	0.0273 (3)	0.6091 (2)	0.0378 (5)	
C7	0.5946 (2)	0.2753 (3)	0.4591 (2)	0.0407 (5)	

### Atomic displacement parameters (Å^2^)

**Table:**

	*U*^11^	*U*^22^	*U*^33^	*U*^12^	*U*^13^	*U*^23^
S	0.0559 (4)	0.0461 (4)	0.0383 (4)	0.0125 (2)	0.0303 (3)	0.0041 (2)
N1	0.0875 (17)	0.0765 (17)	0.0569 (14)	0.0339 (14)	0.0375 (13)	0.0256 (13)
N2	0.0544 (11)	0.0423 (11)	0.0395 (10)	0.0099 (9)	0.0298 (9)	0.0079 (8)
N3	0.0609 (12)	0.0421 (11)	0.0427 (11)	0.0143 (9)	0.0353 (9)	0.0107 (8)
N4	0.0877 (16)	0.0626 (15)	0.0468 (12)	0.0360 (12)	0.0467 (12)	0.0221 (10)
C1	0.0656 (17)	0.0579 (18)	0.078 (2)	0.0224 (13)	0.0426 (16)	0.0289 (14)
C2	0.0675 (17)	0.0420 (15)	0.088 (2)	0.0143 (12)	0.0426 (16)	0.0072 (13)
C3	0.0616 (16)	0.0471 (15)	0.0563 (15)	0.0085 (12)	0.0273 (13)	0.0009 (12)
C4	0.0430 (12)	0.0419 (13)	0.0456 (13)	0.0048 (10)	0.0275 (10)	0.0068 (10)
C5	0.0728 (17)	0.0574 (16)	0.0491 (14)	0.0208 (13)	0.0327 (13)	0.0100 (12)
C6	0.0428 (12)	0.0396 (12)	0.0367 (11)	0.0026 (9)	0.0229 (10)	0.0034 (9)
C7	0.0505 (12)	0.0425 (12)	0.0359 (11)	0.0087 (10)	0.0256 (10)	0.0030 (9)

### Geometric parameters (Å, °)

**Table:**

S—C6	1.743 (2)	C1—C2	1.366 (4)
S—C7	1.736 (2)	C1—H1B	0.9300
N1—C1	1.329 (4)	C2—C3	1.385 (4)
N1—C5	1.335 (3)	C2—H2B	0.9300
N2—N3	1.384 (2)	C3—C4	1.379 (3)
N2—C6	1.289 (3)	C3—H3B	0.9300
N3—C7	1.306 (3)	C4—C5	1.383 (3)
N4—C7	1.342 (3)	C4—C6	1.474 (3)
N4—H4A	0.8600	C5—H5A	0.9300
N4—H4B	0.8600		
			
C7—S—C6	86.65 (10)	C4—C3—H3B	120.5
C1—N1—C5	116.8 (3)	C2—C3—H3B	120.5
C6—N2—N3	113.79 (18)	C3—C4—C5	117.5 (2)
C7—N3—N2	111.67 (17)	C3—C4—C6	122.5 (2)
C7—N4—H4A	120.0	C5—C4—C6	120.0 (2)
C7—N4—H4B	120.0	N1—C5—C4	124.1 (3)
H4A—N4—H4B	120.0	N1—C5—H5A	117.9
N1—C1—C2	123.7 (2)	C4—C5—H5A	117.9
N1—C1—H1B	118.2	N2—C6—C4	124.3 (2)
C2—C1—H1B	118.2	N2—C6—S	113.59 (17)
C1—C2—C3	118.8 (3)	C4—C6—S	122.13 (16)
C1—C2—H2B	120.6	N3—C7—N4	123.6 (2)
C3—C2—H2B	120.6	N3—C7—S	114.30 (16)
C4—C3—C2	119.0 (3)	N4—C7—S	122.07 (16)
			
C5—N1—C1—C2	0.9 (5)	C3—C4—C6—N2	147.8 (2)
N1—C1—C2—C3	−0.9 (5)	C5—C4—C6—N2	−32.8 (3)
C6—N2—N3—C7	0.0 (3)	C3—C4—C6—S	−32.0 (3)
C1—C2—C3—C4	0.2 (4)	C5—C4—C6—S	147.4 (2)
C2—C3—C4—C5	0.5 (4)	C7—S—C6—N2	−0.09 (18)
C2—C3—C4—C6	179.9 (2)	C7—S—C6—C4	179.73 (19)
C1—N1—C5—C4	−0.1 (5)	N2—N3—C7—N4	179.7 (2)
C3—C4—C5—N1	−0.6 (4)	N2—N3—C7—S	−0.1 (3)
C6—C4—C5—N1	180.0 (3)	C6—S—C7—N3	0.10 (19)
N3—N2—C6—C4	−179.8 (2)	C6—S—C7—N4	−179.7 (2)
N3—N2—C6—S	0.1 (3)		

### Hydrogen-bond geometry (Å, °)

**Table:**

*D*—H···*A*	*D*—H	H···*A*	*D*···*A*	*D*—H···*A*
N4—H4A···N3^i^	0.86	2.13	2.959 (3)	163
N4—H4B···N2^ii^	0.86	2.16	3.006 (3)	168

Symmetry codes: (i) −*x*+1, −*y*+1, −*z*+1; (ii) *x*, −*y*+1/2, *z*−1/2.
